# Association of PD‐L1 tumor proportion score ≥20% with early resistance to osimertinib in patients with *EGFR* ‐mutated NSCLC

**DOI:** 10.1002/cam4.6405

**Published:** 2023-08-07

**Authors:** Yusuke Hamakawa, Yoko Agemi, Aya Shiba, Toshiki Ikeda, Yuko Higashi, Masaharu Aga, Kazuhito Miyazaki, Yuri Taniguchi, Yuki Misumi, Yukiko Nakamura, Tsuneo Shimokawa, Yusuke Saigusa, Nobuaki Kobayashi, Hiroaki Okamoto, Takeshi Kaneko

**Affiliations:** ^1^ Department of Respiratory Medicine Yokohama Municipal Citizen's Hospital Kanagawa Japan; ^2^ Department of Biostatistics, Graduate School of Medicine Yokohama City University Yokohama Japan; ^3^ Department of Pulmonology, Graduate School of Medicine Yokohama City University Yokohama Japan

**Keywords:** *EGFR* mutations, EGFR‐TKI, non‐small cell lung carcinoma, osimertinib, programmed cell death‐ligand 1

## Abstract

**Background:**

The relationship between epidermal growth factor receptor tyrosine kinase inhibitor (EGFR‐TKI) resistance, including osimertinib, and programmed cell death‐ligand 1 (PD‐L1) expression status in *EGFR*‐mutated non‐small cell lung carcinoma (NSCLC) remains unclear.

**Patients and Methods:**

We retrospectively analyzed 64 patients with unresectable advanced or metastatic NSCLC carrying *EGFR* exon 19 deletions (ex19del) or *EGFR* exon 21 L858R substitutions (L858R) who received osimertinib as the first‐line treatment. We compared progression‐free survival (PFS) between eligible patients with PD‐L1 tumor proportion scores (TPS) ≥20% and PD‐L1 TPS <20% using the Kaplan–Meier survival plots with a log‐rank test. Multivariate analysis was performed to examine the poor prognostic factors of PFS.

**Results:**

The PD‐L1 TPS ≥20% group included 22 cases (median [range] age: 70.5 [33–86] years; 10 women [45.5%]; 11 current or ex‐smokers [50%]); ECOG performance status (PS) of 0–1/2/3/4 was noted in 16/4/1/1 patients, respectively. The PD‐L1 TPS <20% group included 42 patients (median [range] age 73 [43–88] years; 29 women [69%]; 12 current or ex‐smokers [28.6%]); ECOG PS of 0–1/2/3/4 was noted in 33/6/3/0 cases, respectively. The median PFS was 9.1 and 28.1 months in the PD‐L1 TPS ≥20% and PD‐L1 TPS <20% groups, respectively (log‐rank *p* = 0.013). Multivariate analysis revealed that PD‐L1 TPS ≥20% was associated with PFS (hazard ratio: 2.35, 95% confidence interval: 1.09–5.08, *p* = 0.030).

**Conclusion:**

PD‐L1 TPS ≥20% in patients with *EGFR*‐mutated NSCLC may be associated with early resistance to osimertinib.

## 1 INTRODUCTION

Epidermal growth factor receptor–tyrosine kinase inhibitors (EGFR–TKIs) are widely used as the first‐line systemic treatment for patients with unresectable advanced or metastatic *EGFR*‐mutated non‐small cell lung carcinoma (NSCLC).^1^, ^2^

*EGFR* exon 19 deletions (ex19del) or exon 21 L858R substitutions (L858R), commonly called the *EGFR* mutations because they account for approximately 90% of *EGFR* mutations, are EGFR–TKI‐sensitive mutations.^3^ In contrast, *EGFR* exon 20 T790M substitutions (T790M) are first‐ or second‐generation EGFR–TKI (such as gefitinib or erlotinib)‐resistant mutations.^4^, ^5^

Osimertinib is an irreversible third‐generation EGFR‐TKI with high blood–brain barrier permeability that inhibits EGFR–TKI‐sensitive mutations and T790M‐resistant mutations. The characteristics of osimertinib allow for better outcomes in patients treated with osimertinib than those treated with first‐generation EGFR–TKIs.^6^, ^7^ However, patients with *EGFR* mutations sometimes develop early resistance to osimertinib and little evidence suggests the prognostic factor for osimertinib therapy. An *EGFR* mutation type is known as one of the possible prognostic factors. Studies have shown the difference in prognostic outcomes between ex19del and L858R during osimertinib treatment. An analysis of data from Phase III of the FLAURA trial^7^ comparing osimertinib and gefitinib/erlotinib in patients with common *EGFR*‐mutated unresectable advanced or metastatic NSCLC demonstrated that osimertinib had superior efficacy in NSCLC in patients with ex19del compared to those with L858R.^8^ Although the precise etiological difference of osimertinib efficacy between ex19del and L858R remains unclear, previous evidence indicates that the presence of co‐mutations in key tumor suppressor genes, such as *TP53*, *RB1*, *KEAP1*, *CDKN2A*, or *CTNNB1* affect the difference of osimertinib efficacy between ex19del and L858R.^9^

With the rapid advancement of precision medicine, programmed cell death‐ligand 1 (PD‐L1) expression status in tumor cells has been attracting attention for its relation to the prognostic outcomes associated with EGFR‐TKIs in patients with *EGFR*‐mutated NSCLC. Few studies have shown that prognostic outcomes of first‐generation EGFR–TKIs are unrelated to PD‐L1 expression.^10^, ^11^ On the contrary, some studies have shown that high PD‐L1 expression adversely affects prognostic outcomes in patients receiving first‐generation EGFR–TKIs treatment.^12^, ^13^, ^14^ Regarding third‐generation EGFR–TKIs, some studies have demonstrated that prognostic outcomes of osimertinib are irrelevant to PD‐L1 expression status.^9^, ^15^, ^16^ Contrary to these studies, others have demonstrated that high PD‐L1 expression adversely affects prognostic outcomes in patients receiving third‐generation EGFR–TKIs.^17^, ^18^

Collectively, it remains controversial whether PD‐L1 expression status is associated with prognostic outcomes in patients receiving EGFR–TKIs for *EGFR*‐mutated NSCLC.

Additionally, assuming that PD‐L1 expression status is associated with prognostic outcomes of EGFR–TKIs, there is sparse available evidence on how much PD‐L1 expression affects the prognostic outcomes in patients receiving EGFR–TKIs with *EGFR*‐mutated NSCLC. In particular, the relationship between prognostic outcomes of the third‐generation EGFR–TKI osimertinib and PD‐L1 expression status remains largely unknown. Therefore, we retrospectively assessed whether PD‐L1 expression status affected the prognostic outcomes of osimertinib treatment in patients with *EGFR*‐mutated NSCLC.

## 2 PATIENTS AND METHODS

### 2.1 Study design and patients

This retrospective cohort study examined the relationship between osimertinib resistance and PD‐L1 expression levels in patients with NSCLC with EGFR–TKIs sensitive mutations. We retrospectively analyzed data from 64 pathologically confirmed NSCLC cases (62 cases of adenocarcinoma, 1 adenosquamous carcinoma, and 1 adenocarcinoma with sarcomatoid variant), which were unresectable, advanced, or metastatic, and ex19del‐ or L858R‐positive. These cases were treated with osimertinib as the first‐line treatment, and their tumor tissues were tested for PD‐L1 immunohistochemistry (IHC) at the Yokohama Municipal Citizen's Hospital in Yokohama, Japan between September 2018 and December 2022. To eliminate any potential confounding factors, NSCLC cases that were tested for PD‐L1 IHC in cell blocks from pleural effusions were excluded from the current study due to insufficient evidence.

The treatment included the administration of 80 mg oral osimertinib once daily until disease progression or unacceptable toxicity was identified. We reduced the dose of osimertinib to 40 mg once daily as necessary when low‐grade adverse events occurred. Radiation therapy for central nervous system (CNS) metastasis was performed as necessary before the initiation of osimertinib treatment.

Formalin‐fixed paraffin‐embedded tumor tissue samples were obtained from surgical resections or biopsies. *EGFR* mutations were identified using next‐generation sequencing (Oncomine Dx Target Test Multi‐CDx System®, Thermo Fisher Scientific), monoplex real‐time polymerase chain reaction assays (The cobas® EGFR Mutation Test v2, Roche Diagnostics) or multiplex real‐time polymerase chain reaction assays (The AmoyDx® Multi‐Gene Mutations Detection Kit, Amoy Diagnostics). PD‐L1 expression was identified using IHC assay (anti‐PD‐L1 clone 22C3 pharmDx®, Dako North America). PD‐L1 expression levels were described by tumor proportion score (TPS). The TPS was defined as a ratio of the percentage of the number of PD‐L1 positive tumor cells divided by the total number of PD‐L1 positive tumor cells plus PD‐L1 negative tumor cells.

Data for this study were collected from clinical records and included age at diagnosis; sex; history of smoking; site of metastases; PD‐L1 TPS; Eastern Cooperative Oncology Group performance status (PS); disease stage, based on the Union for International Cancer Control staging system; pathology; and *EGFR* mutation status. Tumor assessment was performed by an investigator in accordance with the Response Evaluation Criteria in Solid Tumors (RECIST) version 1.1.

The study procedures carried out were in accordance with the Declaration of Helsinki and the current ethics guidelines. Ethics approval was obtained from the Yokohama Municipal Citizen's Hospital Ethics Board, which waived the requirement for informed consent owing to the retrospective nature of the study and the blinding of personally identifiable information.

### 2.2 Statistical analysis

The primary endpoint was determined by progression‐free survival (PFS), defined as the period from osimertinib initiation to tumor progression or any‐cause mortality. Patients without progression were censored at the last visit.

We categorized PD‐L1 TPS into low‐, intermediate‐, and high‐expression groups as follows: PD‐L1 TPS <20% as low expression, PD‐L1 TPS ≥20% but <50% as intermediate expression, and PD‐L1 TPS ≥50% as high expression.^19^

We compared PFS among eligible patients with a PD‐L1 TPS ≥20% (intermediate or high expression) and a PD‐L1 TPS <20% (low expression). The PD‐L1 TPS cutoff value was based on a previous, Phase III, open‐label, randomized study of comparison between pembrolizumab and chemotherapy for treatment‐naïve PD‐L1 expressor with unresectable advanced or metastatic NSCLC (KEYNOTE‐042).^19^

Demographic data are presented as proportions or medians. We performed the Wilcoxon signed‐rank test to compare the averages of numerical variables and Fisher's exact test to compare the proportions of qualitative variables between the PD‐L1 TPS ≥20% (intermediate or high expression) and PD‐L1 TPS <20% (low expression) groups.

We compared the PFS of the PD‐L1 TPS ≥20% (intermediate or high expression) group and the PD‐L1 TPS <20% (low expression) group using the Kaplan–Meier method and log‐rank test. Additionally, we conducted exploratory analyses using the Kaplan–Meier method and log‐rank test to compare the PD‐L1 TPS ≥50% (high expression) group to the PD‐L1 TPS <20% (low expression) group, as well as to compare the 20% ≤PD‐L1 TPS <50% (intermediate expression) group to the PD‐L1 TPS <20% (low expression) group, and to compare the PD‐L1 TPS ≥50% (high expression) group to the PD‐L1 TPS <50% (intermediate or low expression) group. Furthermore, using Kaplan–Meier survival plots with a log‐rank test, we conducted an analysis to explore the potential interaction of the PFS between L858R, ex19del, and PD‐L1 TPS, as well as compared the PFS of two distinct patient groups: those with ex19del and PD‐L1 TPS <20% (low expression), and those with the L858R mutation and PD‐L1 TPS ≥20% (intermediate or high expression).

The PD‐L1 TPS cutoff points of 50% were also based on KEYNOTE‐042.^19^

Multivariate analysis using a Cox proportional hazard regression model was performed. We set a maximum of five events per variable for the Cox proportional hazard regression model.^20^ Multivariate analysis was adjusted for age, sex, smoking status, PS ≥2, PD‐L1 TPS ≥20% (intermediate and high expression), L858R substitution, positive CNS metastasis with unstable symptoms, and positive liver metastasis.

The log‐rank test and Cox proportional hazard regression model were evaluated using two‐tailed *p*‐values, and a significance level of *p* < 0.05 was established. All analyses were conducted with EZR (Saitama Medical Center, Jichi Medical University), a modified version of R commander (The R Foundation for Statistical Computing) that includes commonly used biostatistics functions within the R programming language.^21^

## 3 RESULTS

### 3.1 Patient characteristics

Table 1 shows the baseline characteristics of the eligible patients. The PD‐L1 TPS ≥ 20% (intermediate or high expression) and PD‐L1 TPS < 20% (low expression) groups had 22 and 42 patients, respectively. There were no significant differences between the two groups with regard to age, sex, smoking status, PS, the proportion of patients with *EGFR* mutation status, adrenal gland metastasis, bone metastasis, liver metastasis, or CNS metastasis with or without unstable symptoms.

**TABLE 1:** Characteristics of participants (*n* = 64).

Characteristic	PD‐L1 TPS ≥ 20% group	PD‐L1 TPS < 20% group	*p*‐value
	(*n* = 22)	(*n* = 42)	
Median age, years (range)	70.5 (33–86)	73 (43–88)	0.45
Sex			
Men	12 (54.5%)	13 (31%)	0.11
Women	10 (45.5%)	29 (69%)	
Smoking status			
Never	11 (50%)	30 (71.4%)	0.11
Current or ex‐smokers	11 (50%)	12 (28.6%)	
ECOG PS			
0	8 (36.4%)	7 (16.7%)	0.13
1	8 (36.4%)	26 (61.9%)	
2	4 (18.2%)	6 (14.3%)	
3	1 (4.5%)	3 (7.1%)	
4	1 (4.5%)	0 (0%)	
*EGFR* mutation			
Exon 19 deletions	11 (50%)	21 (50%)	1
Exon 21 L858R substitutions	11 (50%)	21 (50%)	
Site of metastasis			
Adrenal gland metastasis	2 (9.1%)	6 (14.3%)	0.70
Bone metastasis	9 (40.9%)	21 (50%)	0.60
Liver metastasis	6 (27.3%)	5 (11.9%)	0.17
CNS metastasis			
with unstable symptoms	1 (4.5%)	5 (11.9%)	0.66
without symptoms	1 (4.5%)	9 (21.4%)	0.14

Abbreviations: CNS, central nervous system; ECOG PS, Eastern Cooperative Oncology Group performance status; *EGFR*, epidermal growth factor receptor.

### 3.2 Survival analysis

**FIGURE 1:**
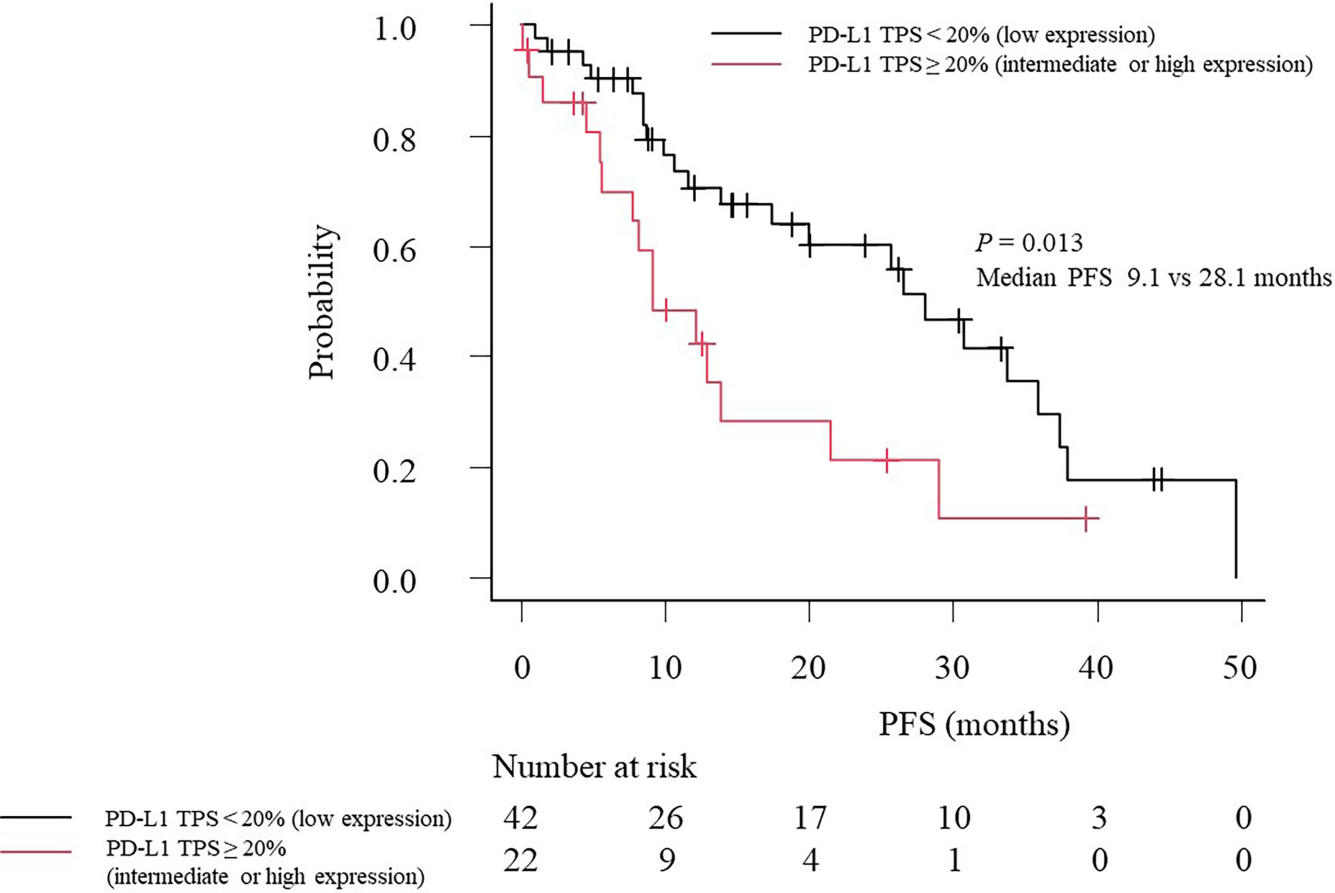
Kaplan–Meier curves of the progression‐free survival (PFS) for programmed cell death‐ligand 1 tumor proportional score (PD‐L1 TPS) ≥20% (intermdiate or high expression) group versus PD‐L1 TPS <20% (low expression) group in patients with epidermal growth factor receptor (*EGFR*)‐mutated non‐small cell lung carcinoma (NSCLC).

The median length of follow‐up was 11.8 months (interquartile range: 6.3–25.5) for all patients. The median PFS in the PD‐L1 TPS ≥20% (intermediate or high expression) and the PD‐L1 TPS <20% (low expression) groups were 9.1 and 28.1 months, respectively. The PFS was significantly shorter in the PD‐L1 TPS ≥20% (intermediate or high expression) group than in the PD‐L1 TPS <20% (low expression) group (log‐rank *p* = 0.013) (Figure 1).

Exploratory analyses were conducted to compare the PFS between the PD‐L1 TPS ≥50% (high expression) and PD‐L1 TPS <20% (low expression) groups, the PFS between the 20% ≤PD‐L1 TPS <50% (intermediate expression) and PD‐L1 TPS <20% (low expression) groups, and the PFS between PD‐L1 TPS ≥50% (high expression) and the PD‐L1 TPS <50% (intermediate or low expression) groups.

**FIGURE 2:**
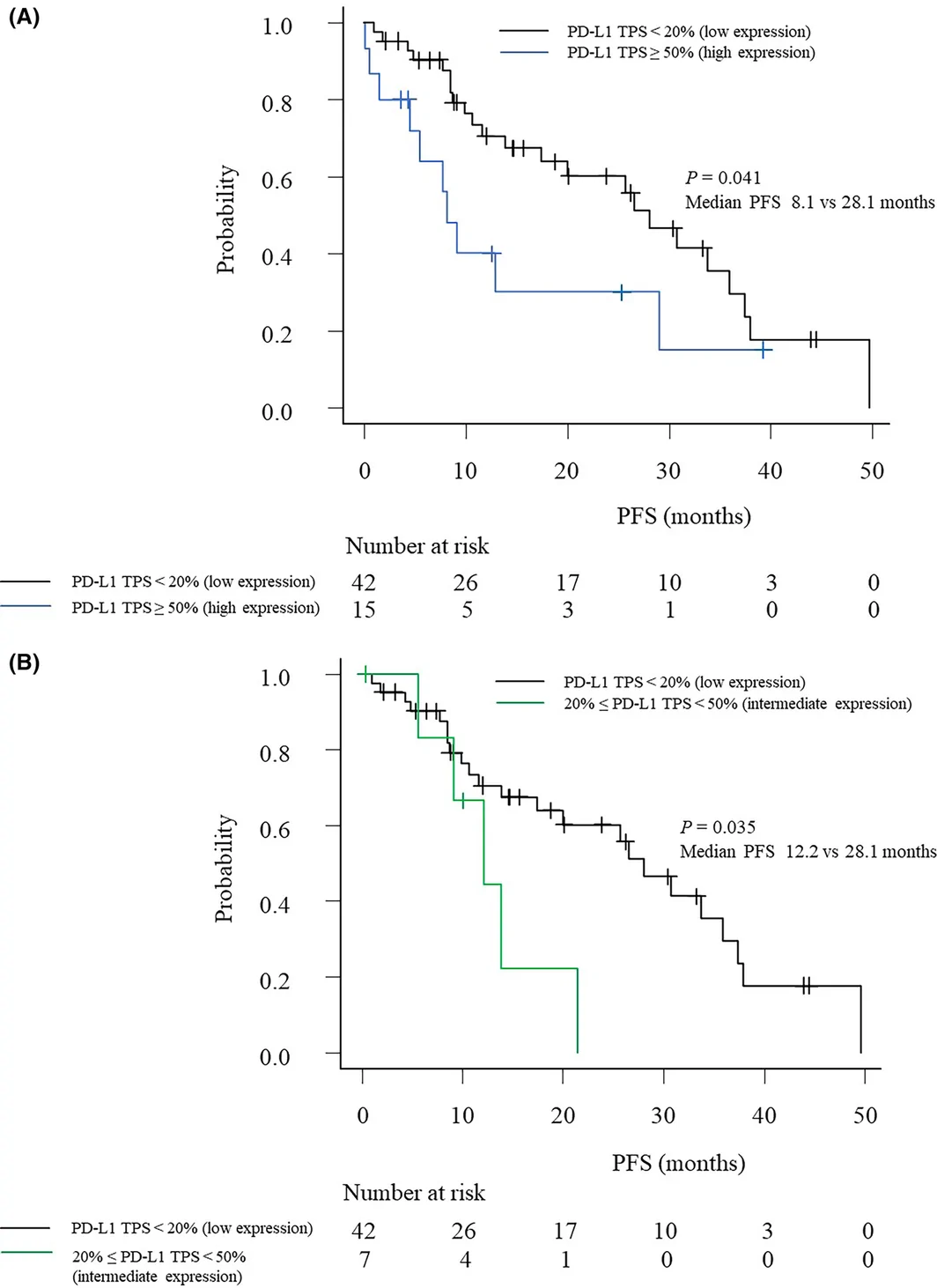
(A) Kaplan–Meier curves of the progression‐free survival (PFS) for programmed cell death‐ligand 1 tumor proportional score (PD‐L1 TPS) ≥50% (high expression) group versus PD‐L1 TPS <20% (low expression) group in patients with epidermal growth factor receptor (*EGFR*)‐mutated non‐small cell lung carcinoma (NSCLC). (B) Kaplan–Meier curves of the progression‐free survival (PFS) for programmed cell death‐ligand 1 tumor proportional score (PD‐L1 TPS) ≥20% and <50% (intermediate expression) group versus PD‐L1 TPS <20% (low expression) group in patients with epidermal growth factor receptor (*EGFR*)‐mutated non‐small cell lung carcinoma (NSCLC).

The median PFS in the PD‐L1 TPS ≥50% (high expression) and the PD‐L1 TPS <20% (low expression) groups were 8.1 and 28.1 months, respectively. The PFS was significantly shorter in the PD‐L1 TPS ≥50% (high expression) group than in the PD‐L1 TPS <20% (low expression) group (log‐rank *p* = 0.041) (Figure 2A).

The median PFS in the 20% ≤PD‐L1 TPS <50% (intermediate expression) and the PD‐L1 TPS <20% (low expression) groups were 12.2 and 28.1 months, respectively. The PFS was significantly shorter in the 20% ≤PD‐L1 TPS <50% (intermediate expression) group than in the PD‐L1 TPS <20% (low expression) group (log‐rank *p* = 0.035) (Figure 2B).

**FIGURE 3:**
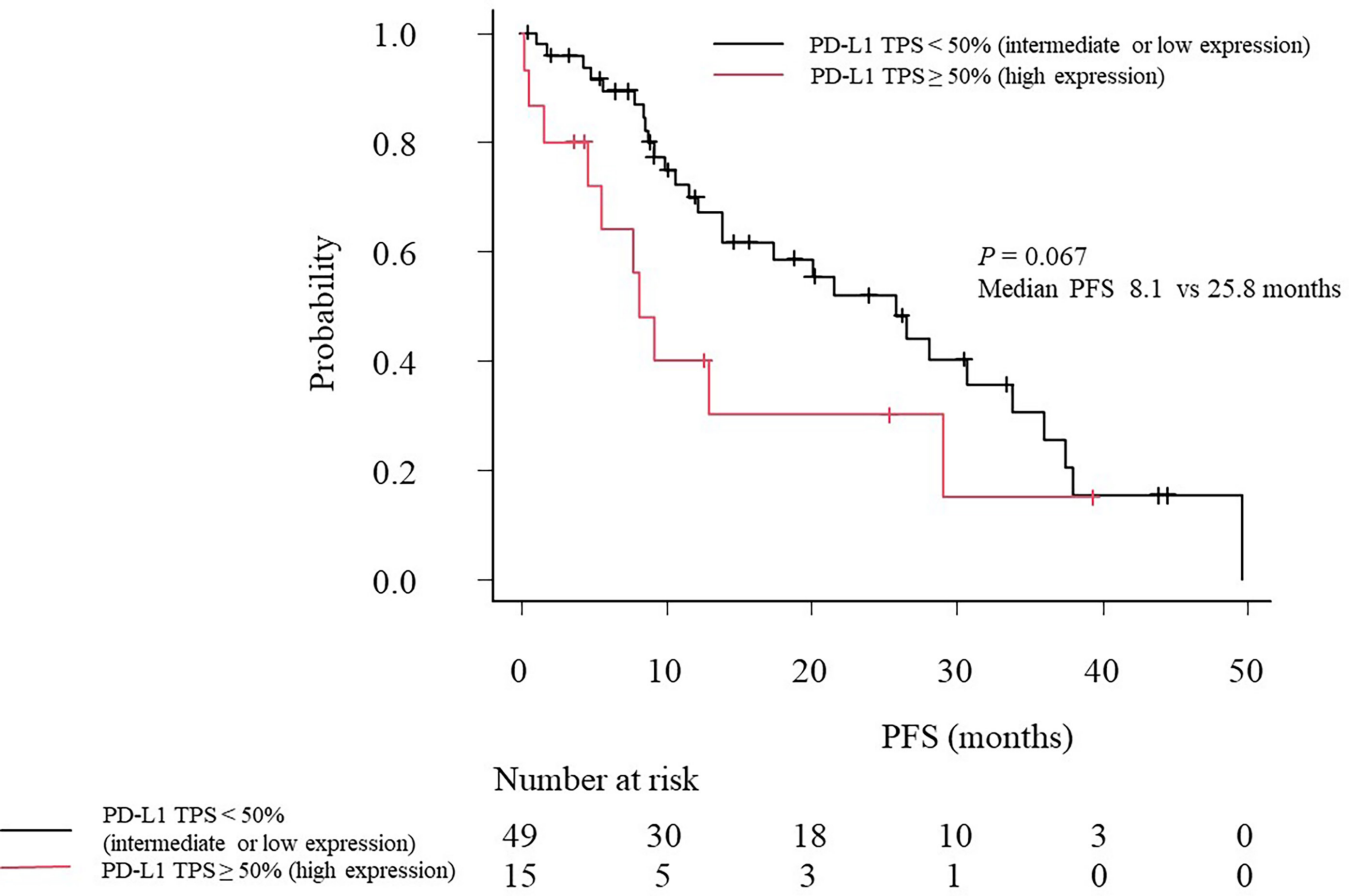
Kaplan–Meier curves of the progression‐free survival (PFS) for programmed cell death‐ligand 1 tumor proportional score (PD‐L1 TPS) ≥50% (high expression) group versus PD‐L1 TPS <50% (intermediate or low expression) group in patients with epidermal growth factor receptor (*EGFR*)‐mutated non‐small cell lung carcinoma (NSCLC).

The median PFS in the PD‐L1 TPS ≥50% (high expression) and the PD‐L1 TPS <50% (intermediate or low expression) groups were 8.1 and 25.8 months, respectively. There were no significant differences in the PFS between the PD‐L1 TPS ≥50% (high expression) group and the PD‐L1 TPS <50% (intermediate or low expression) (log‐rank *p* = 0.067) group (Figure 3).

In addition, we analyzed the interaction between L858R, ex19del, and PD‐L1 TPS, and compared PFS in two patient groups: ex19del and PD‐L1 TPS <20% (low expression), and L858R mutation and PD‐L1 TPS ≥20% (intermediate or high expression).

**FIGURE 4:**
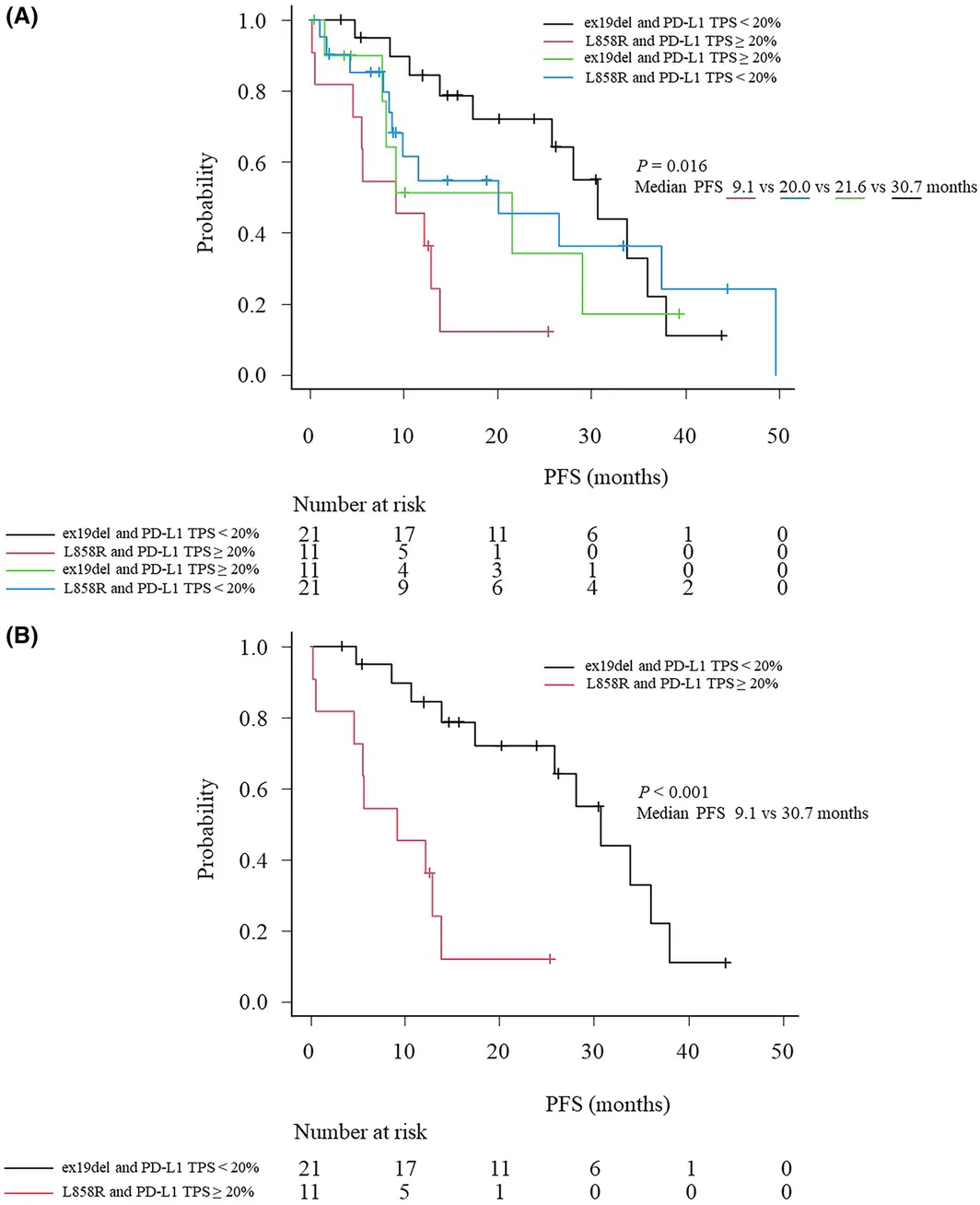
(A) Kaplan–Meier curves were used to compare the progression‐free survival (PFS) in different subgroups of epidermal growth factor receptor (*EGFR*)‐mutated non‐small cell lung carcinoma (NSCLC) based on *EGFR* mutation type (*EGFR* exon 19 deletions [ex19del] or *EGFR* exon 21 L858R substitutions [L858R]) and based on PD‐L1 expression levels (programmed cell death‐ligand 1 tumor proportional score [PD‐L1 TPS] ≥20% [intermediate or high expression] or PD‐L1 TPS <20% [low expression]). (B) Kaplan–Meier curves of the progression‐free survival (PFS) for epidermal growth factor receptor (*EGFR*) exon 19 deletions (ex19del) and programmed cell death‐ligand 1 tumor proportional score (PD‐L1 TPS) <20% (low expression) group versus *EGFR* exon 21 L858R substitutions (L858R) and PD‐L1 TPS ≥20% (intermediate or high expression) group in patients with *EGFR*‐mutated non‐small cell lung carcinoma (NSCLC).

The median PFS was 9.1 months for the L858R and PD‐L1 TPS ≥20% (intermediate or high expression) group, 20.0 months for the L858R and PD‐L1 TPS <20% (low expression) group, 21.6 months for the ex19del and PD‐L1 TPS ≥20% (intermediate or high expression) group, and 30.7 months for the ex19del and PD‐L1 TPS <20% (low expression) group (log‐rank *p* = 0.016) (Figure 4A). The median PFS was 9.1 months for the L858R and PD‐L1 TPS ≥20% (intermediate or high expression) group and 30.7 months for the ex19del and PD‐L1 TPS <20% (low expression) group (log‐rank *p* < 0.001) (Figure 4B).

### 3.3 Prognostic factors

Multivariate analyses revealed that PD‐L1 TPS ≥20% (intermediate or high expression) (hazard ratio [HR]: 2.35 (95% confidence interval [CI]: 1.09–5.08), *p* = 0.030), L858R (HR: 2.26 [95% CI: 1.06–4.83], *p* = 0.035), and presence of positive CNS metastasis with unstable symptoms (HR: 4.21 [95% CI: 1.35–13.07], *p* = 0.013) were prognostic factors for the PFS. Age (HR: 1.00 [95% CI: 0.95–1.04], *p* = 0.84), sex (HR: 0.93 [95% CI: 0.33–2.64], *p* = 0.90), smoking status (HR: 1.56 [95% CI: 0.53–4.61], *p* = 0.42), the PS ≥2 (HR: 1.79 [95% CI: 0.77–4.13], *p* = 0.18), and positive liver metastasis (HR: 2.00 [95% CI: 0.80–5.00], *p* = 0.14) were not significantly associated with the PFS in the multivariate analyses (Table 2).

**TABLE 2:** Cox proportional hazards model for progression‐free survival in all patients.

Factor	Hazard ratio (95% CI)	*p*‐value
Age	1.00 (0.95–1.04)	0.84
Sex		
Female	1	0.90
Male	0.93 (0.33–2.64)	
Smoking status		
Never	1	0.42
Current or ex‐smokers	1.56 (0.53–4.61)	
ECOG PS		
PS <2	1	0.18
PS ≥2	1.79 (0.77–4.13)	
PD‐L1 status		
PD‐L1 TPS <20%	1	0.030
PD‐L1 TPS ≥20%	2.35 (1.09–5.08)	
*EGFR* mutation status		
Exon 19 deletion	1	0.035
Exon 21 L858R substitutions	2.26 (1.06–4.83)	
Liver metastasis		
Negative	1	0.14
Positive	2.00 (0.80–5.00)	
CNS metastasis with unstable symptoms		
Negative	1	0.013
Positive	4.21 (1.35–13.07)	

Abbreviations: CNS, central nervous system; ECOG PS, Eastern Cooperative Oncology Group performance status; *EGFR*, epidermal growth factor receptor; PD‐L1, programmed cell death‐ligand 1; PD‐L1 TPS, programmed cell death‐ligand 1 tumor proportion score.

## 4 DISCUSSION

We demonstrated that intermediate or high PD‐L1 expression (PD‐L1 TPS ≥20%) in patients with *EGFR*‐mutated NSCLC was associated with early resistance to osimertinib.

The PD‐L1 TPS cutoff values of 20% were based on a previous, Phase III, open‐label, randomized study of comparison between pembrolizumab and chemotherapy for treatment‐naïve PD‐L1 expressor with unresectable advanced or metastatic NSCLC (KEYNOTE‐042).^19^

The mechanism of early resistance to osimertinib in the intermediate or high PD‐L1 expression remains unclear. However, one possible explanation is that high‐tumor mutational burden (TMB) leading to osimertinib resistance may be associated with the intermediate or high PD‐L1 expression level in tumor cells. This explanation is based on some previous reports that a higher proportion of PD‐L1 positive tumor cells was observed in high TMB tumors^22^ and that TMB was associated with PD‐L1 tumor cell staining using the IHC assay (PD‐L1 E1L3N XP Rabbit mAb, Cell Signaling Technology) > 20%,^23^ and that high TMB is associated with poor outcomes for targeted therapy in *EGFR*‐mutated lung cancer.^24^ Considering these studies, the PD‐L1 TPS ≥20% group in our study may also have had a high TMB leading to early resistance to osimertinib.

Several other potential mechanisms could lead to early resistance to osimertinib in patients with *EGFR*‐mutated NSCLC with PD‐L1 expression. One such mechanism is the upregulation of Yes‐associated protein 1 (YAP1) by PD‐L1, which induces anti‐apoptosis through a feedback mechanism that involves YAP1/EGFR/extracellular signal‐regulated kinase (ERK)/nuclear factor kappa B (NF‐κB).^25^ Another potential mechanism is that the PD‐L1/B‐cell lymphoma 2 (Bcl2)‐associated athanogene 1 (BAG‐1) axis causes persistent activation of ERK signaling, which leads to resistance to EGFR‐TKIs due to heightened instability of the Bcl‐2 interacting mediator of cell death protein.^26^ Additionally, PD‐L1 may contribute to resistance to EGFR‐TKIs by inducing epithelial‐to‐mesenchymal transition through the activation of the transforming growth factor (TGF‐β)/Smad signaling pathway.^27^

However, additional research is needed to clarify the underlying mechanisms of early osimertinib resistance in intermediate or high PD‐L1 expression.

Limited data exist about comparing prognostic outcomes among patients carrying *EGFR*‐mutated NSCLC with PD‐L1 TPS ≥20% and those with PD‐L1 TPS <20% up to date, although most previous research has compared outcomes using PD‐L1 TPS ≥50%, 50% > PD‐L1 TPS ≥1%, and PD‐L1 TPS <1% as a cut‐off. There is still limited information regarding early resistance to osimertinib in patients with intermediate or high PD‐L1 expression (PD‐L1 TPS ≥20%). To the best of our knowledge, this is the first study to demonstrate that PD‐L1 TPS ≥20% may have a significantly negative impact on prognosis in patients receiving osimertinib treatment.

Although a previous study demonstrated a significant difference in the PFS between the PD‐L1 TPS ≥50% and PD‐L1 TPS <50% groups,^17^, ^18^ our study and another previous study have shown no difference in prognostic outcomes between the PD‐L1 TPS ≥50% and PD‐L1 TPS <50% groups.^16^ The reason for the inconsistency of the results is that we acknowledge that there may be other factors influencing the outcomes, such as the presence of intermediate PD‐L1 expression (20% ≤PD‐L1 TPS <50%) having an impact on a poor prognosis in the PD‐L1 TPS <50% group, as well as the potential impact of compound mutations associated with resistance to osimertinib^28^ on the inconsistency of the results. In regard to compound mutation, the response rate of osimertinib was found to be only 27% for patients with compound mutations that included T790M.^28^ Additionally, both PFS and overall survival were significantly shorter in this group of patients. Given that the proportion of compound *EGFR* mutations was identified to be 14% among all mutations,^29^ their presence may contribute to the inconsistency of the results.

Our finding that intermediate or high PD‐L1 expression (PD‐L1 TPS ≥20%) in patients with *EGFR*‐mutated NSCLC was a factor contributing to shorter PFS of osimertinib is meaningful for therapeutic strategy. Initially, when treating patients carrying *EGFR*‐mutated NSCLC with intermediate or high PD‐L1 expression, it is important to assess osimertinib efficacy at brief intervals owing to the potential for early resistance to osimertinib. Second, early switching to immune checkpoint inhibitors should be actively considered when a patient's general condition worsens. When a patient's general condition deteriorates, but their tumor assessment does not meet the criteria for RECIST‐defined progressive disease, it can be difficult to determine whether this is due to the clinical progression of the disease or some kind of adverse events. In this situation, it can be challenging to decide whether to continue treating with osimertinib because of its proven efficacy in the FLAURA trial.^7^ In such cases, early switching to immune checkpoint inhibitor treatments may be worth giving careful consideration for patients carrying *EGFR*‐mutated NSCLC with intermediate or high PD‐L1 expression, due to the potential for early resistance to osimertinib.

L858R and positive CNS metastasis with unstable symptoms were also found to have an association with the PFS of osimertinib. The finding that L858R had shorter PFS was consistent with previous studies.^8^ It is notable that our analysis to explore the potential interaction between L858R, ex19del, and PD‐L1 TPS indicated that the combination of ex19del and PD‐L1 TPS <20% had a more pronounced effect on prognosis compared to the combination of L858R and PD‐L1 TPS ≥20%. Regarding CNS metastasis with unstable symptoms, it is uncertain whether there is an association between positive CNS metastasis with unstable symptoms and prognostic outcome due to the exclusion of patients with unstable symptomatic CNS metastasis from the FLAURA trial.^7^ Our study showed that CNS metastasis with unstable symptoms was a prognostic factor contributing to shorter PFS of osimertinib. Regarding liver metastasis, although it has been reported as a poor prognostic factor for first‐ or second‐generation EGFR–TKIs in *EGFR*‐mutated NSCLC patients,^30^ our study found no significant association between liver metastasis and the PFS of third‐generation EGFR–TKI osimertinib. The relationship between the presence of liver metastasis in patients with *EGFR*‐mutated NSCLC and the prognostic outcome of osimertinib remains unclear and requires further investigation.

The present study has several limitations. First, the retrospective nature, single‐center design, and small sample size of the study could have led to unintentional selection bias. Further prospective studies are required to validate our results. Second, our analysis did not consider the presence of compound mutations in *EGFR*‐mutated NSCLC, as next‐generation sequencing was approved by the Pharmaceuticals and Medical Devices Agency in Japan only in June 2019, whereas our study was conducted in September 2018. A previous study has shown that compound *EGFR* mutations are associated with osimertinib resistance.^28^ Hence, resistance to osimertinib in our patients may have been affected by unknown compound *EGFR* mutations. Consequently, confounding factors could not be excluded. Therefore, future research should explore and focus on compound *EGFR* mutations.

## 5 CONCLUSION

Our findings suggest that *EGFR*‐mutated NSCLC with PD‐L1 TPS ≥ 20% may have early resistance to osimertinib (shorter PFS compared to those with PD‐L1 TPS <20%).

## AUTHOR CONTRIBUTIONS

**Yusuke Hamakawa:** Conceptualization (lead); data curation (lead); formal analysis (lead); investigation (lead); methodology (lead); project administration (lead); resources (equal); visualization (lead); writing – original draft (lead); writing – review and editing (lead). **Yoko Agemi:** Conceptualization (equal); resources (equal); supervision (lead); writing – review and editing (equal). **Aya Shiba:** Resources (equal); writing – review and editing (equal). **Toshiki Ikeda:** Resources (equal); writing – review and editing (equal). **Yuko Higashi:** Resources (equal); writing – review and editing (equal). **Masaharu Aga:** Resources (equal); writing – review and editing (equal). **Kazuhito Miyazaki:** Resources (equal); writing – review and editing (equal). **Yuri Taniguchi:** Resources (equal); writing – review and editing (equal). **Yuki Misumi:** Resources (equal); writing – review and editing (equal). **Yukiko Nakamura:** Resources (equal); writing – review and editing (equal). **Tsuneo Shimokawa:** Resources (equal); writing – review and editing (equal). **Yusuke Saigusa:** Formal analysis (lead); methodology (equal); writing – review and editing (equal). **Nobuaki Kobayashi:** Conceptualization (equal); methodology (equal); supervision (equal); writing – review and editing (equal). **Hiroaki Okamoto:** Conceptualization (equal); methodology (equal); resources (equal); supervision (lead); writing – review and editing (lead). **Takeshi Kaneko:** Conceptualization (equal); methodology (equal); supervision (lead); writing – review and editing (lead).

## CONFLICT OF INTEREST STATEMENT

The authors in this study declare that they have no conflicts of interest.

## ETHICS STATEMENT

Ethics approval was obtained from the Yokohama Municipal Citizen's Hospital Ethics Board, which waived the requirement for informed consent owing to the retrospective nature of the study and the blinding of personally identifiable information.

## Data Availability

The data that support the findings of this study are available on request from the corresponding author. The data are not publicly available due to privacy or ethical restrictions.
